# Mosquito-Parasite Interactions Can Shape Filariasis Transmission Dynamics and Impact Elimination Programs

**DOI:** 10.1371/journal.pntd.0002433

**Published:** 2013-09-12

**Authors:** Sara M. Erickson, Edward K. Thomsen, John B. Keven, Naomi Vincent, Gussy Koimbu, Peter M. Siba, Bruce M. Christensen, Lisa J. Reimer

**Affiliations:** 1 Department of Pathobiological Sciences, University of Wisconsin, Madison, Wisconsin, United States of America; 2 Center for Global Health and Diseases, Case Western Reserve University, Cleveland, Ohio, United States of America; 3 Papua New Guinea Institute of Medical Research, Madang, Papua New Guinea; 4 Department of Entomology, Michigan State University, East Lansing, Michigan, United States of America; 5 Papua New Guinea Institute of Medical Research, Goroka, Papua New Guinea; National Institute of Allergy and Infectious Diseases, United States of America

## Abstract

The relationship between mosquito vectors and lymphatic filariasis (LF) parasites can result in a range of transmission outcomes. Anophelines are generally characterized as poor vectors due to an inability to support development at low densities. However, it is important to understand the potential for transmission in natural vectors to maximize the success of elimination efforts. Primary vectors in Papua New Guinea (n = 1209) were dissected following exposure to microfilaremic blood (range 8–233 mf/20 µl). We examined density dependent and species-specific parasite prevalence, intensity and yield, barriers to parasite development as well as impacts on mosquito survival. We observed strikingly different parasite prevalence and yield among closely related species. Prevalence of infective stage larvae (L3s) ranged from 4.2% to 23.7% in *An. punctulatus*, 24.5% to 68.6% in *An. farauti s.s.* and 61.9% to 100% in *An. hinesorum* at low and high density exposures, respectively. Injection experiments revealed the greatest barrier to parasite development involved passage from the midgut into the hemocoel. The ratio of L3 to ingested mf at low densities was higher in *An. hinesorum* (yield = 1.0) and *An. farauti s.s.* (yield = 0.5) than has been reported in other anopheline vectors. There was a negative relationship between mosquito survival and bloodmeal mf density. In *An. farauti s.s.*, increased parasite yield and survival at low densities suggest greater competence at low microfilaremias. In Papua New Guinea the likelihood of transmission will be strongly influenced by vector composition and changes in the mf reservoir as a result of elimination efforts. Global elimination efforts will be strengthened by the knowledge of transmission potential in the context of current control measures.

## Author Summary

Lymphatic filariasis (LF) elimination requires interrupting transmission of microfilaria (mf) from humans to mosquitoes for 5–7 years, the average life span of adult worms. Current mf prevalence thresholds, below which transmission cannot be sustained, are unknown. Anopheline-transmitted LF is thought to be easily eliminated following community-wide distribution of anthelminthic drugs, based on this genera's poor vectorial ability. We observed up to a 30-fold difference in parasite yield in experimentally infected mosquitoes of the *Anopheles punctulatus* group, the primary vectors in Papua New Guinea. In two species, *An. farauti* and *An. hinesorum*, prevalence and intensity of infection were higher than what has previously been described for anopheline mosquitoes. Differences in vector competence were largely attributable to a failure of microfilaria to survive escape of the midgut. Other barriers to parasite development include damage upon ingestion and, to a lesser degree, melanization. These results challenge the assumption that anophelines are poor vectors and provide further insight as to why mass drug administration alone has been unsuccessful in stopping LF transmission in PNG. Large differences in vector competence among closely related species indicate that transmission thresholds will be site and vector specific, and control efforts should be tailored accordingly.

## Introduction

Human lymphatic filariasis (LF) is a mosquito-borne disease that is a leading cause of morbidity worldwide. 1.4 billion people in 81 countries are at risk of infection with the nematode parasites *Wuchereria bancrofti*, *Brugia malayi* or *B. timori*. Clinical manifestations, including acute fevers, chronic lymphedema, elephantiasis and hydrocele, result in the loss of 5.9 million disability-adjusted life-years [1]. Even individuals with mild manifestations are stigmatized in their societies and suffer psychological impacts [2]. *W. bancrofti* parasites, which account for 90% of the global disease burden, dwell in the lymphatic system, where the adult female worms release microfilariae (mf) into the blood. Mf are taken up in the blood meal of a mosquito, and go through several developmental stages within permissive vector species. Infective-stage larvae (L3s) actively escape from the mosquito mouthparts during a bloodfeeding event and enter a new vertebrate host through skin.

Infection prevalence and morbidity is on the decline worldwide due to mass drug administration (MDA) of anthelminthic drugs coordinated by the Global Program to Eliminate Lymphatic Filariasis (GPELF). These single dose regimens target mf in the bloodstream, and therefore prevent transmission to mosquitoes. However, in order to reach the goal of elimination by the year 2020, numerous challenges must be overcome. Elimination of LF requires annual MDA with high coverage and compliance for at least 5 years in order to interrupt transmission through the lifespan of adult worms [3], [4], [5], a difficult undertaking in light of logistical and financial constraints. Perhaps most importantly, thresholds for transmission cessation are currently unknown and are site-specific. Therefore, program managers currently lack the necessary tools to make informed decisions about when to stop, scale-up or reinstate MDA.

Current transmission cessation thresholds are based on dominant vector genera [6], due to differences in vector-parasite relationships [7]. Culicine vectors are generally regarded as efficient vectors of LF, with proportionally greater output of L3s as the number of mf ingested decreases (limitation). In contrast, anophelines have been characterized as inefficient vectors, with proportionally lesser output of L3s as fewer mf are ingested (facilitation) [8]. For this reason, it has been hypothesized that stopping transmission by reducing the mf reservoir with MDA can be more easily attained in areas where LF is anopheline-transmitted [9]. However this paradigm may not extend to all anophelines. In Papua New Guinea, where the prevalence of LF is among the highest in the world [10], members of the *Anopheles punctulatus* group are the primary vectors but MDA has been unsuccessful in stopping transmission [11], [12], [13]. This contradiction suggests that critical appraisal of local mosquito vectors is needed to enable better estimations of intervention endpoints and enhance predictive transmission modeling algorithms [14].

As interventions are employed to control LF, whether by MDA or vector-based interventions, it becomes increasingly important to demonstrate the influence of a decreasing mf reservoir on the vector-parasite relationship. The aim of this study was to determine the vector competence of individual species within the *An. punctulatus* group to *W. bancrofti* in the context of specific mf densities. This research was performed within a critical timeframe, in an endemic country with ongoing and future plans for MDA-based control of LF [15] as well as a large-scale distribution of insecticide-treated bed nets [16]. Both interventions hold promise to interrupt transmission of LF in PNG, either by reducing human microfilaremia, reducing vector biting rates, or interfering with host-seeking at times of maximum mf density in the host peripheral blood [13]. However, predicting the long-term impact of these campaigns remains difficult without an understanding of the vector-parasite relationships in this highly endemic country.

## Materials and Methods

Institutional review boards at the Papua New Guinea Institute of Medical Research (PNGIMR) and the University of Wisconsin-Madison (UW-Madison), as well as the Papua New Guinea Medical Research Advisory Committee (MRAC), reviewed and approved the inclusion of human subjects in this research (PNGIMR IRB No. 1008; UW-Madison IRB M-2010-1158; MRAC No. 10.46). All study participants were recruited non-continuously between September 2010 and October 2012 and provided prior written informed consent. Antihelminthic drugs (DEC and albendazole) were provided to study communities by the PNG Department of Health.

### Mosquito collections and maintenance

*Anopheles* larvae were collected from temporary pools in the Madang, Sumkar and Usino Bundi Districts of Madang Province and the Dreikikir-Ambunti district of East Sepik Province, PNG. Colonized *An. farauti* s.s., originating from East New Britain Province, PNG in 1967, was used for the majority of exposures for this species because comparative studies showed no difference in infection prevalence or mean intensity between colony and wild *An. farauti s.s.* (Table 1)

**Table 1:** Development of *Wuchereria bancrofti* in colony and wild-caught *Anopheles farauti* s.s. from PNG.

Microfilaremia (mf/20 µl blood)	Source of *An. farauti*	Total dissected^a^	Infection Prevalence (95% CI)	Total worms recovered^a^	Mean instensity (95% CI)
35	Colony	161	30.4 (23.5, 38.2)^b^	112	2.0 (1.6, 2.4)^c^
	Reared from wild larvae	32	25.0 (12.2, 42.3)^b^	16	2.0 (1.4, 3.3)^c^

a >1 day post-exposure.

b Infection prevalence compared by Unconditional Test, exact p-value = 1.0.

c Mean Intensity of developing parasites compared by Bootstrap t-test, p-value = 0.9415.

Mosquitoes were maintained in an insectary with a 12∶12 light cycle that included a 30-minute crepuscular period for dawn and dusk. Natural temperatures in this environment ranged from 27–28°C, in the afternoon, to 23.5°C at night. The relative humidity was increased by placing damp towels on top of each cage or carton (∼85% RH inside colony cages). Larvae were reared in plastic pans with water collected from a local creek, and fed finely ground Tetramin™ in solution. Adult mosquitoes were provided 20% sucrose solution *ad libitum* via cotton pledgets. Species identification was performed after inclusion in an experiment and prior to dissection for the recovery of parasites. If the adults were reared from wild-caught larvae, then morphological characteristics were observed with a stereomicroscope and used to classify individuals into the three major *An. punctulatus* morpho groups (*An. punctulatus*, *An. farauti* s.l. and *An. koliensis*) prior to dissection. These characteristics include proboscis coloration and presence or absence of the wing sector spot [17]. In addition, the legs of each individual mosquito were collected, coded, and stored for species confirmation by PCR-RFLP of the ITS2 region [18]. A portion of individuals from the *An. farauti* s.s. colony was also verified.

### *W. bancrofti* exposures

Adults (≥18 years of age) were recruited as study volunteers from suspected LF endemic villages in Madang and East Sepik Provinces. Individuals providing informed consent to participate in the study were initially screened for the presence or absence of *W. bancrofti* circulating antigen using BinaxNow© Filariasis rapid card tests (Alere Inc., Waltham, MA). Subsequently, antigen positive volunteers were asked to provide a venous blood sample, which was collected after 22:00 and transported to the laboratory. A compound microscope with phase contrast optics was used to quantify the number of mf per 20 µl blood in a 2% formalin wet mount. Microfilaremia was confirmed in triplicate and the remaining blood was used for feeding mosquitoes via water-jacketed membrane feeders fitted with parafilm or pig intestine membranes [19]. Sucrose-starved, female *An. punctulatus* (2–7 days old) and *An. farauti* s.s. (3–6 days old) were allowed to feed on microfilaremic blood from 11–26 hours post collection according to mosquito feeding preferences. Mf motility was observed by wet mount at the time of feeding. Fully engorged mosquitoes were sorted from non-fed and partially fed females and maintained in the insectary for up to 18 DPE.

### Mosquito dissections to recover and observe parasites

**Figure 1:**
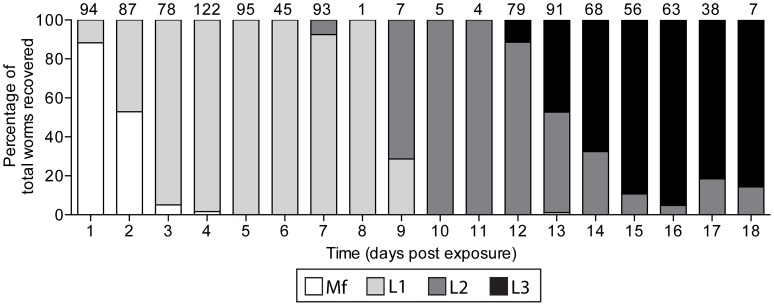
The development of *Wuchereria bancrofti* from microfilaria to infective-stage larvae in *Anopheles farauti* s.s. The number of parasites observed at each timepoint is listed above the bar.

The timing of mosquito dissections was based on average *W. bancrofti* development times (Figure 1). Mosquitoes were cold anesthetized and divided into body regions in separate drops of *Aedes* saline [20] for dissection and parasite recovery. Mosquito tissues were teased apart with 0.15-mm insect pin probes, cover-slipped and examined using phase-contrast optics. Additionally, between 3 and 24 hours post engorgement, a portion of mosquitoes' midguts were removed and lysed in distilled water. Slides were dried overnight before methanol fixation and Giemsa staining. They were microscopically examined to quantify the number of mf ingested and the degree of damage, caused by the cibarial armature,following ingestion. Later in parasite development (12–18 DPE), the body regions were dissected in a drop of saline and worms were observed with a stereomicroscope with dark-field backlighting. Mosquitoes were individually tracked to record morphological identification, molecular species confirmation, parasite prevalence and intensity, mf damage and melanization of worms.

### Isolation and intrathoracic inoculation of microfilariae

To better determine the influence blood feeding and the midgut environment might have on parasite survival, intrathoracic inoculations were used to place mf directly in the hemocoel. Microfilariae were isolated from blood samples using syringe tip filtration devices, fitted with 5 µM membranes (Millipore Isopore TMTP) and chilled *Aedes* saline solution. Mf were rinsed off each filter with 1–2 ml of saline solution in conical vials, and spun at 1,000 rpm for 10 min at 4°C to concentrate the parasites and remove most of the fluid. A single drop of saline containing concentrated parasites was transferred to a microscope slide and mf were loaded into finely pulled glass capillary needles for injection into mosquitoes. A dissection microscope was used to observe the loading of approximately 10–20 mf per needle for mosquito injections. Mosquitoes were cold anesthetized for 3 minutes at −20°C immediately prior to the injection procedure. Anesthetized mosquitoes were injected with mf in a minimum volume (0.5–1.0 µl) of *Aedes* saline, into a membranous cuticle area on the lateral side of the mesothorax [21]. Mosquitoes that survived for >12 hours post inoculation were dissected and developmental stage of recovered parasites was observed.

### Data analysis

Microfilaria densities used in this study represent natural infection levels and were categorized as low (<50 mf/20 uL), medium (50–100 mf/20 uL) and high (>100 mf/20 uL) for the study communities. Mosquito infection is summarized by the prevalence of infection with 95% CI (adjusted Wald/Sterne's interval) and mean intensity (total number of recovered parasites divided by total number of infected mosquitoes) with 95% CI (Bootstrap BCa). To compare the prevalence of parasite infection, Fisher's Exact tests were performed for comparisons of 3–6 populations and unconditional exact tests to compare the prevalence of infection between two populations. Bootstrap t-tests were performed to compare mean intensities. All between species comparisons were done on *An. punctulatus* and *An. farauti s.s.* Because sample sizes were too low in *An. hinesorum* for statistical analyses, only intensity and prevalence are presented for illustrative purposes.

Quantitative Parasitology 3.0, a freeware program, was used for statistical analysis (http://www.zoologia.hu/qp/qp.html) and GraphPad Prism (version 5.0d) for generating graphs and figures.

## Results

From one to 18 days post ingestion of *W. bancrofti*-infected blood via membrane feeders, *An. farauti* s.s. (n = 652), *An. punctulatus* (n = 505) and *An. hinesorum* (n = 52) were dissected to recover and observe parasites. The details of each exposure are available in Table S1.

### Numbers of parasites ingested

**Figure 2:**
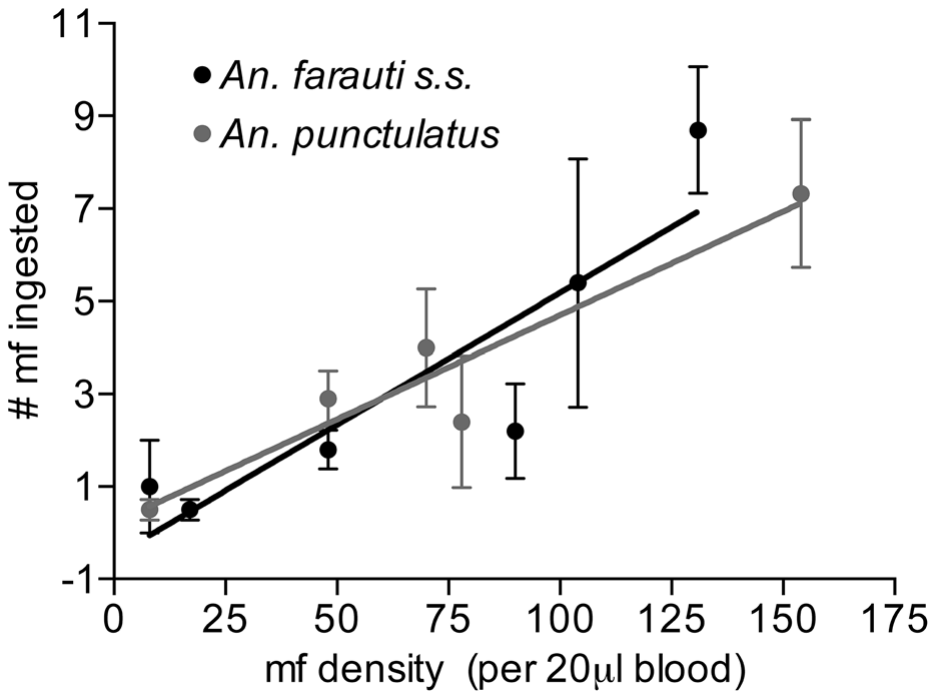
Relationship between host mf density and the number of mf ingested. Regression equations are not significantly different from each other (*An. punctulatus* Y = 0.045*X+0.22, *An. farauti* Y = 0.057*X-0.51; Slope: p = 0.5; Intercept: p = 0.89).

To investigate potential differences in the number of parasites ingested by each species following bloodfeeding on a range of microfilaremias, a portion of mosquitoes were dissected immediately (<18 hours) following the feeds and mf were counted. Total mf recovery revealed a linear relationship between the number of mf ingested and the density of mf in the bloodmeal at the ranges studied (Figure 2). There was no significant difference in the mean number of mf ingested between *An. punctulatus* and *An. farauti s.s.* (ANCOVA p = 0.6).

### Prevalence and intensity

The prevalence and mean intensity of *W. bancrofti* in experimentally infected PNG anophelines is presented in Table 2. Infection prevalence and intensities were calculated for both the number of developing worms (any stage) recovered after 1 DPE and infective-stage larvae only. In both *An. punctulatus* and *An. farauti s.s.*, the infectious bloodmeal parasitemia had a significant effect on the prevalence of developing and infective- stage larvae (Fisher's exact p<0.001 for each). There was also a significant difference in the prevalence of developing worms between the two species within mf densities (low p<0.001, med p<0.003, high p<0.013). The mean intensity of developing worms was significantly higher in *An. farauti s.s.* as compared with *An. punctulatus* (p = 0.0015).

**Table 2:** Prevalence and intensity of *W. bancrofti* infection in mosquitoes exposed to microfilaremic blood.

		All developing parasites (mf-L3; from 1.5–18 DPE)		Infective-stage larvae (L3s from 13.5 DPE)		
	Microfilaremia^a^ [total feeds]	Prevalence % (95% CI) [total dissected]	Mean intensity (95% CI)[total worms]	Prevalence (95% CI) [total dissected]	Mean Intensity (95% CI) [total L3s]	Parasite Yield^b^ (Mean L3/Mean mf ingested)
*An. punctulatus*	Low [3]	8.5 (5.3,13.27)[200]	1.2 (1.0,1.4)[21]	4.2 (0.37,14.8)[48]	1.0 (n.a.)[2]	0.03 (0.04/1.55)
	Med [2]	17.7 (13.0,23.6)[198]	1.8 (1.4,2.3)[63]	0 (n.a.)[14]	n.a.	0 (0/4.62)
	High [2]	31.0 (21.4,42.5) [71]	4.0 (2.7,5.6)[88]	23.7 (12.8,39.4)[38]	2.8 (1.6, 5.2)[25]	0.07 (0.67/9.18)
*An. farauti* s.s.	Low [4]	28.9 (24.3,34.0)[342]	2.0 (1.7,2.2)[197]	24.5 (19.5,30.5)[232]	2.0 (1.7, 2.4)[116]	0.50 (0.5/1.0)
	Med [2]	48.7 (38.8,9.6)[78]	4.3 (3.3,6.2)[164]	37.0 (21.5,55.8)[27]	3.1 (2.0, 4.4)[31]	0.23 (1.15/4.93)
	High [2]	79.2 (72.8,84.5)[183]	4.7 (4.1,5.3)[683]	68.6 (51.9,81.6)[35]	3.3 (2.5, 4.4)[80]	0.24 (2.29/9.50)
*An. hinesorum*	Low [2]	64.3 (44.0,77.4)[29]	2.4 (1.9,3.0)[44]	61.9 (40.8,79.3)[21]	2.2 (1.7,2.5)[29]	1 (1.38/0.91)
	Med [1]	100 (51.1,100)[5]	6.4 (1.0,11.8)[32]	0 (n.a.)[1]	n.a.	n.a.
	High [1]	85.7 (46.7,99.5)[7]	5.2 (2.2,9.3)[31]	100 (29.0,100)[2]	7.5 (n.a.)[15]	0.94 (7.50/8.00)

a Microfilaremia: Low (8–48 mf/20 µl blood), Medium (70–97 mf/20 µl blood), High(130–233 mf/20 µl blood).

b As defined by Pichon *et al.* 1974 for comparisons with historical data.

### Parasite yield

**Figure 3:**
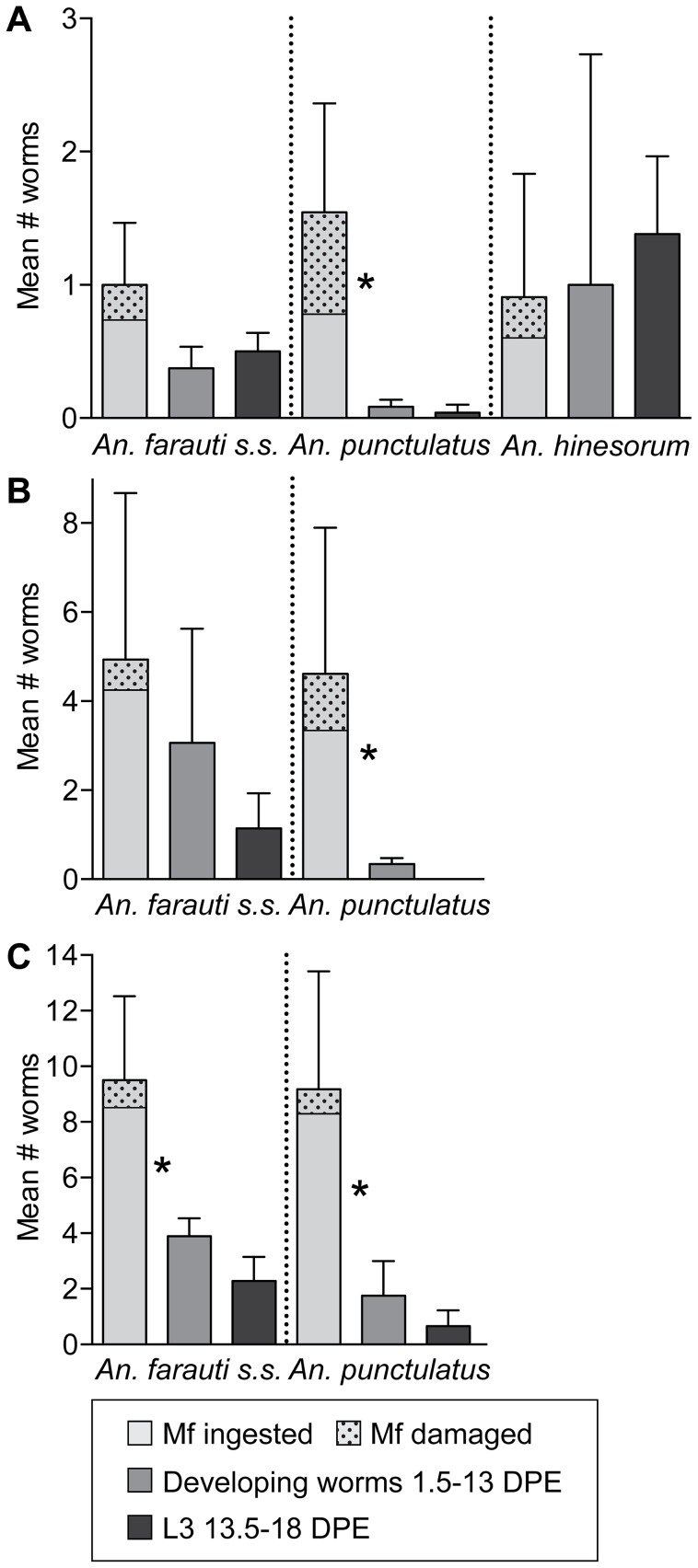
Attrition of developing *W.* *bancrofti* in multiple anopheline species from A) low B) medium and C) high microfilarial density blood. The mean number of worms ingested (95% CI), including the proportion that were damaged upon ingestion by the cibarial armature, and the relative yield of developing worms (any stage, between 1 and 13 DPE) or the yield of L3s (between 13.5–18 DPE). Non-parametric t test compares mean number of intact mf with the mean number of developing worms, and the mean number of developing worms with the mean number of L3s (other stages present beyond 13.5DPE are not included in the mean). Bonferonni adjusted alpha for multiple comparisons = 0.004 *p<0.0001.

There was a significant decrease in the mean number of developing worms (>1DPE) compared to the mean number of intact mf in the midgut and body (<1DPE) recovered from *An. punctulatus* at all densities and *An. farauti* s.s. at high density only (P<0.0001, Figure 3). There was no significant difference between the mean number of worms recovered from 1.5 through 13 DPE and the mean number of L3s recovered from 13.5 through 18 DPE. The limited data from *An. hinesorum* suggests attrition through each developmental stage is minimal. The ratio of L3s to the number of mf ingested is presented in Table 2. This ratio is highest in *An. hinesorum*, ranging from 1.0 to 0.94, and lowest in *An. punctulatus*, ranging from 0.03 to 0.07 at low and high densities, respectively.

Potential barriers to *W. bancrofti* development were investigated. A greater proportion of mf were damaged following ingestion of a low density as compared with a high density microfilaremic bloodmeal in both *An. punctulatus* and *An. farauti s.s.* (p<0.001 and p = 0.03 respectively). At low microfilaremias, a greater proportion of damaged mf were observed in *An. punctulatus* than in *An. farauti* s.s. and *An*. *hinesorum*, but at high microfilaremic bloodmeals the number of damaged mf was comparable between *An. punctulatus* and *An. farauti* s.s. (Figure 3).

**Figure 4:**
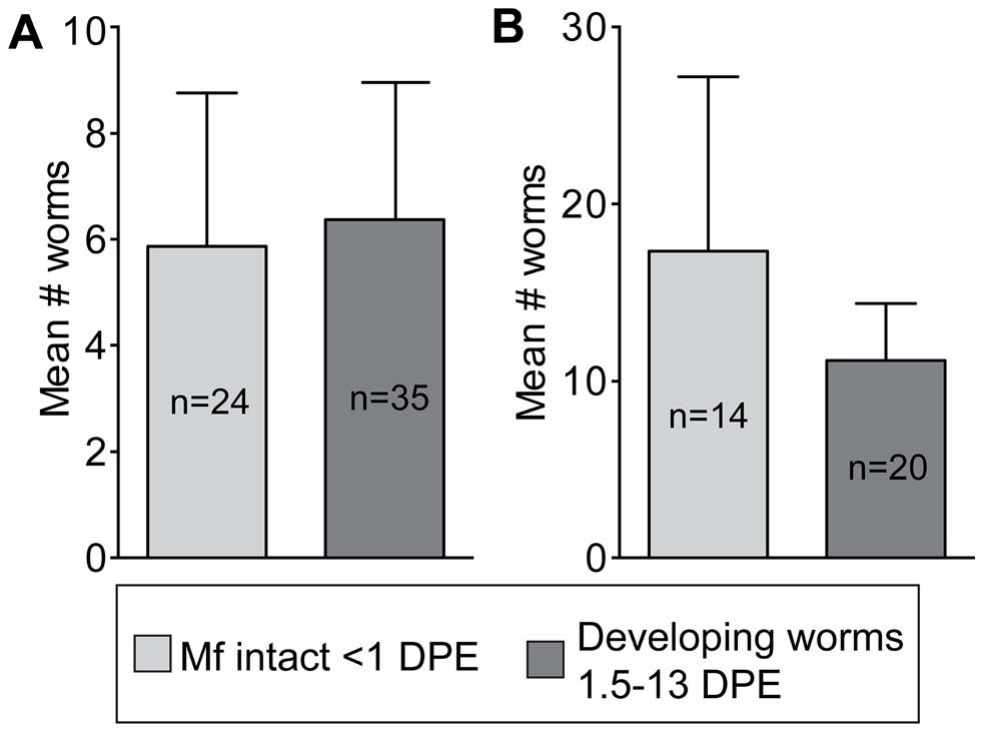
Attrition of developing *W.* *bancrofti*, incoluated into the hemocoel of A) *An. farauti* and B) *An. punctulatus*. There was no significant difference between the mean number of parasites recovered immediately post-injection (<1 day) and the mean number of developing worms (recovered from 1.5–13 days post-injection).

To test the hypothesis that the high degree of attrition observed in *An. punctulatus* is attributable to early developmental barriers (ingestion and/or the mosquito midgut environment), mf were introduced directly into the hemocoel, effectively by-passing the midgut. When mf were intrathoracically inoculated, there was no difference in the number of live mf recovered immediately post-injection and developing worms (Figure 4).

Melanization was observed in one *An. farauti s.s.* exposed to a low density infection. In this individual one L2 was partially covered in melanin. Melanization was observed in one *An. punctulatus* and one *An. farauti* s.s. that had received mf via injection. In both cases a single mf was fully melanized. Melanized sheaths were observed in the hemocoel of both species indicating *W. bancrofti* exsheathment can occur after traversing the midgut.

### Mosquito survivorship

**Figure 5:**
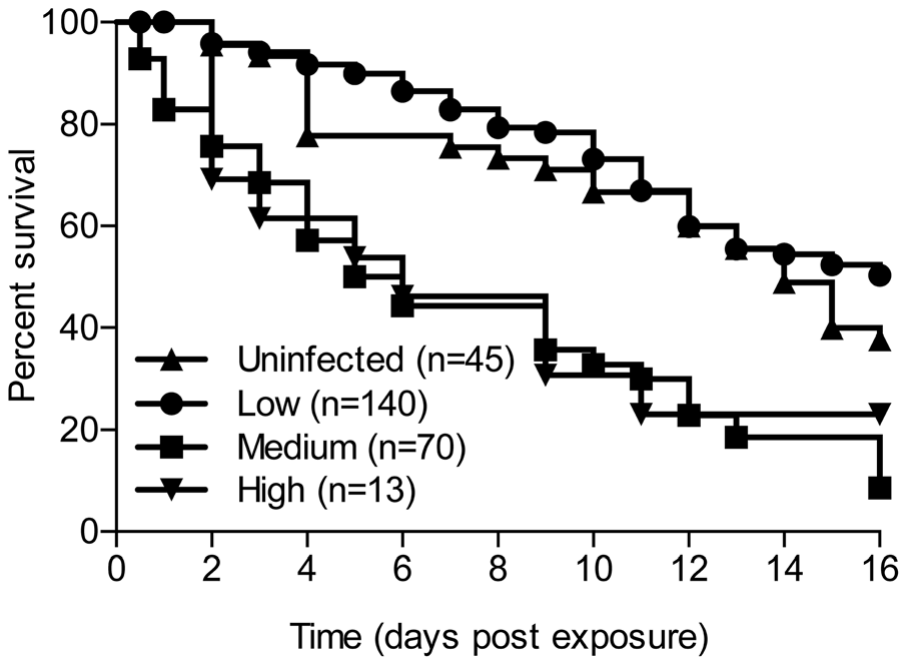
Survival curves for *An.* *farauti s.s*. following exposure to different densities of microfilaremic blood.

Infection with *W. bancrofti* had a negative impact on survivorship in *An. farauti* s.s (Figure 5) and mortality was correlated with the density of infection. No difference was observed in survival between mosquitoes exposed to uninfected blood compared to low density microfilaremia. Survival 14 days post exposure was 60% in mosquitoes exposed to low density microfilaremia and only 20% in mosquitoes exposed to medium and high densities.

## Discussion

In this study, we assessed the vector competence of members of the *Anopheles punctulatus* group to *W. bancrofti*. Overall, the prevalence and intensity of parasite infection in mosquitoes, and the proportion of damage to mf upon ingestion, were observed to all be density-dependent. However, not all examined species supported parasite development to the same degree. Some measures of infection, including (1) overall prevalence and intensity, (2) prevalence and intensity of infective-stage larvae, and (3) parasite yield (i.e., proportion of mean L3s produced from number of parasites ingested), were strikingly different at comparable mf densities between closely related species. *An. hinesorum* is incriminated as a vector of *W. bancrofti* here for the first time and our results show that this species is highly competent. Although less abundant than *An. punctulatus* and *An. farauti s.s*. in our study sites, this species is ubiquitous in both the inland and coastal regions of PNG, and abundant south of the central range [22].

Current assumptions regarding the inability of anophelines to transmit filariasis at low density microfilaremias may not extend to all vectors, as evidenced by comparing our results to previous studies that employed similar methodology. The *An. farauti s.s.* parasite yield is five times higher than what has been reported in African anopheline LF vectors at low density parasite exposures, including *An. gambiae*, *An. arabiensis*, *An. funestus*, *An. melas* and *An. merus* [8]. In addition, the mean number of L3s produced at medium and high mf density feeds is higher than any other anopheline vector, as reviewed in Snow et al. [7].

Compared to *An. farauti s.s*., a greater proportion of *An. punctulatus* fail to support filarial worm development. The greatest reduction in prevalence occurs at 1 DPE, which corresponds to the time that microfilaria traverse the midgut epithelium. This attrition was not observed when mf were introduced directly into the hemocoel. These results suggest that the reduced vector competence of *An. punctulatus* is attributable to the midgut barrier. Although the cibarial armature causes some damage to mf, the degree of damage at high densities is comparable to the amount of damage in *An. farauti* and cannot explain the difference in prevalence between the two species at medium and high densities.

Very few mosquitoes harbored melanized *W. bancrofti* in this study, a result that differs from a previous study that observed nearly 50% of infected *An. punctulatus* had elicited some degree of melanization response [23]. Differences in the observed melanization phenotypes may be related to differences in midgut microbiota [24], acquired from the larval environment or differences in reactive oxygen species (ROS). ROS are associated with melanotic encapsulation [25], [26] and could be elevated due to environmental stress [27], or inhibited by the anticoagulant and anti-oxidant heparin [28].

The question of whether mosquito survivorship is adversely effected by *W. bancrofti* infection is paramount in estimating vector competence, yet relatively few studies [29] have addressed this issue. We have shown convincingly that *W. bancrofti* infection and parasite intensity influence mosquito survivorship. We found increased mortality in *An. farauti s.s*. that ingested blood with a medium or high density of mf relative to low mf density blood and uninfected controls. In *An. punctulatus*, previous studies have found that there was no difference in mortality between low and high density feeds [29]. Tissue damage, which may or may not lead to mosquito death, is often observed when development of second-stage larvae (L2s) is completed in the thorax and the actively motile L3s relocate to the body and head of the mosquito [30], [31]. Although greater impacts on survival would be expected in the more competent vector this is not always the case with naturally occurring parasite-mosquito interactions. Co-evolution of parasite-host interactions has likely selected a minimal consequence of infection on host survivorship. This is evidenced by observations of certain mosquito vectors eliciting a minimal immune-related or damage repair response following intracellular filarial worm development, e.g., *Mansonia uniformis* and *Armigeres subalbatus* infected with *Brugia malayi* and *B. phangi* respectively [30], [32].

In vectors such as *An. farauti s.s.* that are highly susceptible, increased mortality at high density infections will reduce the potential for transmission in the field because these mosquitoes may not survive the extrinsic incubation period (EIP). Alternatively, as microfilaremia decreases in the population, the transmission potential may increase. In *An. farauti s.s*, the significantly higher survivorship through the EIP at low density coupled with increased parasite yield could result in higher vectorial capacity. This observation challenges the assumption that anophelines are incapable of transmitting LF at a low microfilaremia.

This study demonstrates a linear relationship between vertebrate host mf densities and mean number of mf ingested, which corresponds roughly to the number of mf we would expect in 1 µL of blood. However, previous studies [33] have observed a concentrating effect at low host mf densities (<10 mff/mL), which was below the threshold for inclusion in the present study. The effect of mf concentration at low densities warrants further investigation, especially as MDA campaigns continue to decrease the reservoir of mf in endemic communities.

Efforts to eliminate lymphatic filariasis through mass drug administration are underway in Papua New Guinea. In addition, the nationwide distribution of long-lasting insecticidal nets is a part of the National Department of Health Malaria Control Program. Both campaigns hold promise for the elimination of *W. bancrofti* transmission by reducing the prevalence of mf in the human population, reducing vector biting rates, or interfering with mosquito biting at times of peak microfilaremia. The success of such programs hinges on the ability to reach worm breakpoint levels (the human mf prevalence below which transmission cannot be sustained). Our research suggests that estimated thresholds will be different between the two primary vectors and elimination may be more achievable in the inland and lowland regions where *An. punctulatus* is most abundant. Other studies have also found sympatric species of the *An. gambiae* complex and M and S molecular forms [34], [35] to have different competency to transmit *W. bancrofti*. Further research on the vector competence of primary LF vectors around the world, in the context of a diminishing mf reservoir, is needed in order to maximize the success of the Global Programme to Eliminate Lymphatic Filariasis. Furthermore, models for LF transmission cessation should be catered to geographic region and control efforts must respond accordingly.

## Supporting Information

Table S1 **The number of mosquitoes dissected and worms recovered for each exposure.**
(DOCX)
