# Supplementary material for: Mosquito-Parasite Interactions Can Shape Filariasis Transmission Dynamics and Impact Elimination Programs
Source: PLoS Negl Trop Dis. 2013 Sep 12;7(9):e2433. doi: 10.1371/journal.pntd.0002433 (PMC3772046; doi:10.1371/journal.pntd.0002433)
Supplement: Table S1 — The number of mosquitoes dissected and worms recovered for each exposure. (DOCX) [file pntd.0002433.s001.docx]

Supplemental Table 1. The number of mosquitoes dissected and worms recovered for each exposure

|  |  |  |  |  | dissections for developing worms >1 DPE | | | dissections for infective larvae >13DPE | | |
| --- | --- | --- | --- | --- | --- | --- | --- | --- | --- | --- |
|  | Density category | Microfilaremia† (mean mf/20uL) | Total mosq dissected | No. mosq dissected <1 DPE | Total mosq dissected (1-18DPE) | No. positive mosquitoes | total worms recovered | No. mosq dissected >13DPE | No. positive mosquitoes | total L3 recovered |
| *colony An. farauti* s.s. | Low | 17 | 41 | 10 | 31 | 8 | 12 | 23 | 3 | 6 |
|  | Low | 35 | 163 | 2 | 161 | 49 | 96 | 150 | 35 | 70 |
|  | Low | 48 | 87 | 10 | 77 | 30 | 65 | 29 | 12 | 31 |
|  | Medium | 97 | 82 | 10 | 72 | 38 | 164 | 24 | 10 | 31 |
|  | High | 131 | 180 | 15 | 165 | 136 | 626 | 30 | 21 | 73 |
|  | High | 233 | 13 | 0 | 13 | 8 | 47 | 3 | 3 | 7 |
| *wild An. farauti* s.s. | Low | 8 | 29 | 0 | 29 | 1 | 2 | 2 | 0 | 0 |
|  | Low | 17 | 11 | 0 | 11 | 2 | 4 | 0 | 0 | 0 |
|  | Low | 35 | 34 | 2 | 32 | 8 | 16 | 28 | 7 | 9 |
|  | Low | 48 | 1 | 0 | 1 | 1 | 2 | 0 | 0 | 0 |
|  | Medium | 70 | 2 | 0 | 2 | 0 | 0 | 0 | 0 | 0 |
|  | Medium | 97 | 4 | 0 | 4 | 0 | 0 | 3 | 0 | 0 |
|  | High | 131 | 0 | 0 | 0 | 0 | 0 | 0 | 0 | 0 |
|  | High | 233 | 5 | 0 | 5 | 1 | 10 | 2 | 0 | 0 |
| *wild An. punctulatus* | Low | 8 | 170 | 0 | 170 | 13 | 16 | 27 | 0 | 0 |
|  | Low | 35 | 9 | 0 | 9 | 1 | 1 | 6 | 1 | 1 |
|  | Low | 48 | 33 | 12 | 21 | 3 | 4 | 15 | 1 | 1 |
|  | Medium | 75 | 207 | 14 | 193 | 33 | 60 | 12 | 0 | 0 |
|  | Medium | 97 | 5 | 0 | 5 | 2 | 3 | 2 | 0 | 0 |
|  | High | 154 | 64 | 10 | 54 | 16 | 51 | 33 | 8 | 24 |
|  | High | 233 | 17 | 0 | 17 | 6 | 37 | 5 | 1 | 1 |
| *wild An. hinesorum* | Low | 35 | 1 | 0 | 1 | 0 | 0 | 1 | 0 | 0 |
|  | Low | 48 | 39 | 11 | 28 | 18 | 44 | 20 | 13 | 29 |
|  | Medium | 97 | 5 | 0 | 5 | 5 | 32 | 1 | 0 | 0 |
|  | High | 233 | 7 | 0 | 7 | 6 | 31 | 2 | 2 | 15 |
| *† Exposures of different species using the same mf density were conducted on the same blood sample* | | | | | | | | | |  |
